# On the shape and likelihood of oceanic rogue waves

**DOI:** 10.1038/s41598-017-07704-9

**Published:** 2017-08-15

**Authors:** Alvise Benetazzo, Fabrice Ardhuin, Filippo Bergamasco, Luigi Cavaleri, Pedro Veras Guimarães, Michael Schwendeman, Mauro Sclavo, Jim Thomson, Andrea Torsello

**Affiliations:** 1 Institute of Marine Sciences, Italian National Research Council (ISMAR-CNR), Venice, 30122 Italy; 2 Univ. Brest, CNRS, IRD, Ifremer, Laboratoire d’Océanographie Physique et Spatiale (LOPS), IUEM, Plouzané, France; 3 DAIS – Università Ca’ Foscari, Venice, Italy; 4 Applied Physics Laboratory, University of Washington, Seattle, Washington, USA; 5 Ecole Centrale de Nantes, LHEEA lab – UMR6598, Nantes, France

## Abstract

We consider the observation and analysis of oceanic rogue waves collected within spatio-temporal (ST) records of 3D wave fields. This class of records, allowing a sea surface region to be retrieved, is appropriate for the observation of rogue waves, which come up as a random phenomenon that can occur at any time and location of the sea surface. To verify this aspect, we used three stereo wave imaging systems to gather ST records of the sea surface elevation, which were collected in different sea conditions. The wave with the ST maximum elevation (happening to be larger than the rogue threshold 1.25*H*_s_) was then isolated within each record, along with its temporal profile. The rogue waves show similar profiles, in agreement with the theory of extreme wave groups. We analyze the rogue wave probability of occurrence, also in the context of ST extreme value distributions, and we conclude that rogue waves are more likely than previously reported; the key point is coming across them, in space as well as in time. The dependence of the rogue wave profile and likelihood on the sea state conditions is also investigated. Results may prove useful in predicting extreme wave occurrence probability and strength during oceanic storms.

## Introduction

There is increasing consensus^1–3^ that a likely physical mechanism explaining the formation of oceanic rogue waves in stormy conditions is the spatio-temporal focusing due to the dispersive nature of water waves in intermediate-deep waters^4–6^, further enhanced by second-order non-resonant nonlinearities^7–9^. The role of the modulation instability due to third-order nonlinearities on the statistics of ocean waves^10–12^ was shown to have a minor effect during directionally spread sea states^13–15^. The same conclusion holds for the extreme wave temporal profile^1^. Here we do not consider other physical mechanisms, such as the presence of ocean currents or, in shallow waters, the bottom topography that may cause wave energy to focus^16^ in a small area.

Whatever the physical mechanism that leads to energy concentration (we shall discuss this point later), rogue waves appear as an erratic 3D phenomenon that can occur at any time and location of the sea surface^5, 17^. However, the majority of available instruments records time series of the values of the sea surface elevation collected at a single point. Accordingly, random amplitudes and phases turn out to be frequency dependent only, and the directional spreading is not resolved. We mention, but do not discuss here, the tendency of wave buoys to underestimate the crest height of individual high waves^18^, which compounds the problem of observing rogue waves from buoy time series. As a consequence, point-like instruments provide only a “tunnel vision” of rogue waves, as the occasional presence of those waves as outliers within a time series is due to the dynamical effects of a coherent and large 3D wave group that focuses nearby the specific observational point^19, 20^. In this respect, with reference to the iconic Draupner rogue wave event^21^, Cavaleri *et al*.^22^ argue that rogue waves are relatively common and part of the realm of stormy 3D waves: the key point is coming across them. This is possible using observational systems (e.g. via stereo wave imaging)^23, 24^ or numerical simulations (e.g. via high-order spectral calculations of the Euler equations for water waves)^25^ capable of capturing the temporal evolution of unsteady 3D wave fields over a sea surface region.

In this study, we interpret rogue waves as space-time (ST) maxima, and we use five ST records of sea surface elevations collected with different stereo wave imaging systems to reveal key aspects of the rogue waves behavior, in particular their temporal profile and probability of occurrence, in connection, also, with the sea state conditions. These aspects were investigated by other scholars^2, 26–31^, but a verification using global maxima of ST wave fields is missing. The main goal of the present paper is to strengthen the rogue wave framework in a multi-dimensional observational and statistical context, in order to show that rogue waves are indeed a likely event in very different storm conditions.

This paper is organized as follows. Firstly, we provide the characteristics of the sea states and of the observed rogue waves, of which we analyze the shape and likelihood. Secondly, the link between the rogue wave events and the sea state parameters is examined. We conclude discussing the consequence of our analysis in the framework of rogue wave studies.

## Results

### Observation of rogue waves in space and time

**Figure 1:**
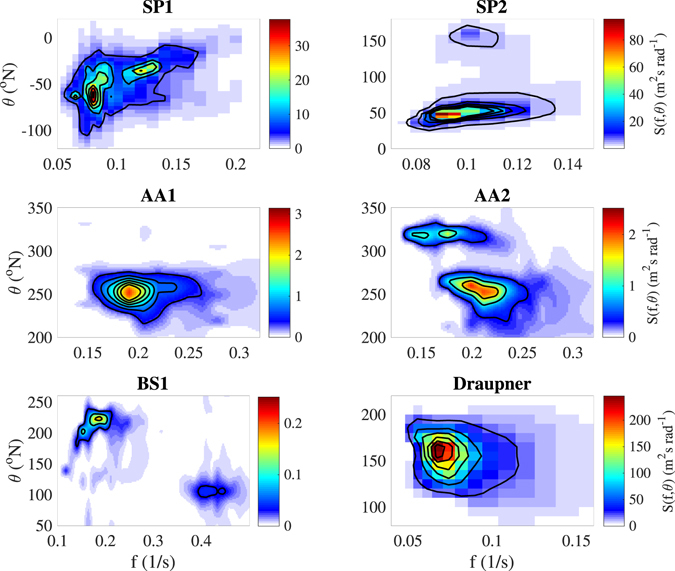
Frequency (*f*) - direction (*θ*) wave spectrum *S* during the stereo acquisitions, and for the Draupner case (throughout the paper we use flow direction of the wave energy). *Station P* spectra (SP1 and SP2) were derived from the directional WaveRider buoy parameters; *Acqua Alta* (AA1 and AA2) and Black Sea (BS1) spectra were derived from stereo data using the extended maximum entropy principle method. The Draupner spectrum was computed by the wave model ECWAM at 14-km resolution forced with ECMWF winds at 9-km resolution^22^. Given the wide range of energy levels, axis limits and color-scale change among panels.

**Figure 2:**
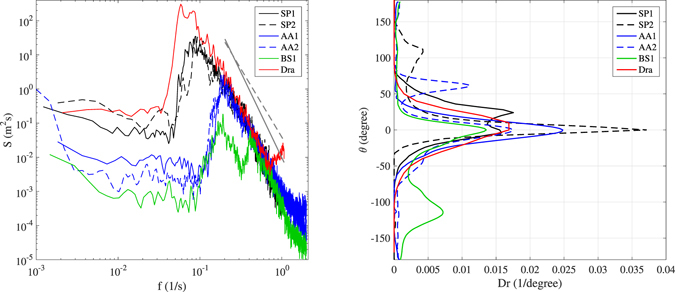
(left panel) Omni-directional frequency spectrum *S*(*f*). The dashed and solid gray lines are reference spectral slopes proportional to *f* ^−4^ and *f* ^−5^, respectively. (right panel) Omni-frequency directional distribution (*Dr*) of the wave energy around the peak direction of the spectrum. As for the Draupner case, the frequency spectrum was computed from the measured time record, whereas the directional distribution was derived from the modeled spectrum.

In this study, we investigate five ST wave records collected using three different stereo wave imaging systems deployed at stations in the Pacific Ocean (near *Station P*), Adriatic Sea (*Acqua Alta* platform), and Black Sea (*Katsiveli* platform) during active wind conditions. The examined sea states have both uni- and bi-modal distribution of energy (Figs 1 and 2), with significant wave height *H*_s_ ranging from about 0.5 m to 4.6 m (Table 1). The omni-directional frequency *f* spectrum *S*(*f*) for each record is shown in Fig. 2 (left panel), including also the modeled spectrum at the time of the Draupner rogue wave event^3^. Albeit three out of the five directional spectra (namely SP2, AA2 and BS1 in Fig. 1) show a well defined bi-modality of the energy distribution, only the waves within the record BS1 present two clearly distinct modes in the spectrum *S*(*f*) at frequencies about 0.18 Hz and 0.41 Hz (left panel of Fig. 2). All energy distributions show an equilibrium range with slope proportional to *f* ^−4^, in accordance, for example, with the Kolmogorov-type energy cascade caused by the resonant four wave-interactions^32^, or the model of the equilibrium range by Phillips^33^.

**Table 1:** Date and location of the wave observations (labeled as SP1, SP2, AA1, AA2, BS1) with the stereo systems.

	Date (UTC)	Location	*H*_s_ (m)	*T*_p_ (s)	*T*_z_ (s)	*z*_m_ (m)	*γ*_RW_	*A* (m^2^)	*D* (s)	*N*_3D_
SP1	2014/12/28, 19:29	Pacific Ocean (*Station P*)	4.64	11.4	7.8	6.50	1.40	940	3200	655
SP2	2015/01/05, 20:30	Pacific Ocean (*Station P*)	3.86	10.7	7.2	5.51	1.43	940	1740	433
AA1	2014/03/10, 09:40	Adriatic Sea (*Acqua Alta*)	1.34	5.4	3.6	2.12	1.59	2893	1798	10830
AA2	2014/03/27, 09:10	Adriatic Sea (*Acqua Alta*)	1.36	5.2	3.7	2.17	1.60	2893	3333	11853
BS1	2013/09/26, 11:45	Black Sea (*Katsiveli*)	0.48	5.3	2.6	0.64	1.34	378	1798	11492
Dra	1995/01/01, 15:00	North Sea (*Draupner plat*.)	11.9	14.4	10.6	18.5	1.55	-	1200	—

Wave parameters: *H*_s_ is the significant wave height, *T*_p_ the spectral peak period, *T*_z_ the average zero-crossing period, *z*_m_ the maximum sea surface elevation within the ST region spanning the sea surface area *A* and the duration *D*. *N*_3D_ is the theoretical number of individual 3D waves^34–36^ over the ST region of volume *AD*. The rogue wave strength is given by the ratio *γ*_RW_ = *z*_m_/*H*_s_. For the Draupner case (labeled as Dra), *z*_m_ = max{*z*(*t*) | *t* ∈ *D*}, in so far as only a time series *z*(*t*) was recorded at the platform.

**Figure 3:**
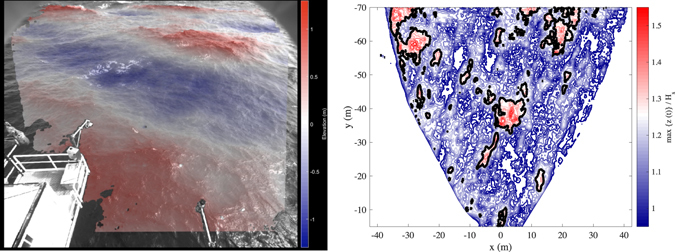
(left panel) Example of 3D wave field (shown in the camera reference system) measured with a stereo wave imaging system installed on the *Acqua Alta* platform (northern Adriatic Sea, Italy). (right panel) The contour lines show the normalized maximum sea surface elevation max{*z*(*t*) | *t* ∈ *D*}/*H*_s_ at each position *P* ∈ *Ω* during the time interval *D*. The thick black contour corresponds to the rogue wave threshold 1.25*H*_s_.

**Figure 4:**
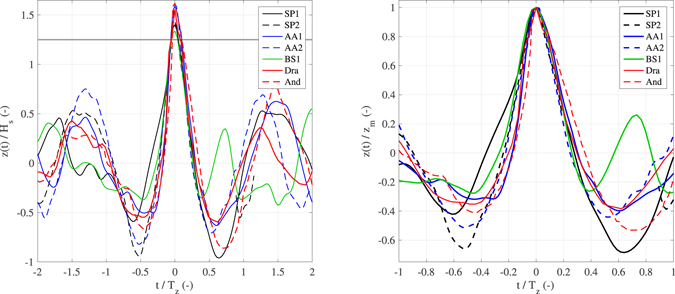
(left panel) Normalized (with *H*_s_ and *T*_z_) temporal profile of sea surface elevations around the observed maximum crest height (*z*_m_) of the ST records. Time of the maximum crest is set equal to zero for all profiles. The horizontal gray line at 1.25 represents the rogue threshold. Note that for *t* > 0 some data are missing in the record SP2. (right panel) Normalized (with *z*_m_ and *T*_z_) temporal profile of sea surface elevations around the observed maximum crest height (*z*_m_) of the ST records. Axis limits change between panels for the sake of clarity. The Draupner (Dra) and Andrea (And) rogue waves are also shown.

Typical result of the stereo-photogrammetric processing is a 3D wave field (Fig. 3) representing the sea surface elevation *z* over an horizontal region *Ω* ∈ ℜ^2^ with area *A*, i.e. *z*(*x*, *y*), where ***x*** = (*x*, *y*) denotes the coordinate vector. The collection of multiple fields stacked up over the time span *D* of the stereo-image acquisition produces a ST record of sea surface elevations, i.e. a function *z* = *z*(*x*, *y*, *t*), spanning both spatial and temporal domains. In search of rogue waves we avoid to detect the largest wave groups^37^, but instead we search the global maximum sea surface elevation *z*_m_ = max{*z*(*x*, *y*, *t*) | (*x*, *y*) ∈ *Ω*, *t* ∈ *D*} within each ST record. This global maximum is considered as one realization of the random variable “maximum crest height”. The marginal temporal profile *z*(*t*) of the maximum waves is extracted from *z*(*x*, *y*, *t*) at the position (*x*_0_, *y*_0_) ∈ *Ω* where *z* = *z*_m_ (Fig. 4). All these maximum waves are rogue (Table 1) as they meet the classical geometric criterion *z*_m_ > 1.25*H*_s_^38^, with roguish strength *γ*_RW_ = *z*_m_/*H*_s_ ranging between 1.34 (record BS1) and 1.60 (record AA2). The crest-to-trough height *H*_m_ of the waves is larger than 2.2*H*_s_ (*H*_m_ > 2.2*H*_s_ is an alternative criterion to characterize rogue waves) except for the record BS1, for which *H*_m_ = 1.7*H*_s_. For comparison, the rogue wave measured at the Draupner platform on 01/01/1995 has *z*_m_ = 1.55*H*_s_ and *H*_m_ = 2.15*H*_s_^21, 39^.

### The temporal profile of rogue waves

For all ST rogue waves, including the Draupner event, Fig. 4 shows the actual temporal profiles *ad hoc* scaled with the corresponding integral parameters of the sea state *H*_s_ and average zero-crossing period *T*_z_ (on the left panel), and with the local wave parameter *z*_m_ (on the right panel). The waves preceding and following the central one of the groups exhibit relatively small and random elevations, which begin to be narrowly concentrated around a deterministic profile for *z* > *H*_s_ (or about 0.65*z*_m_). Indeed, the difference among the rogue wave profiles between the instants crossing the level *z* = *H*_s_ is in the order of 2% for the rising face and 5% for the falling face of the characteristic wave periods. We note that for elevations *z* > *H*_s_ all profiles are asymmetric with respect to *z*_m_, and positively skewed, that is the front steepness is higher. This is also the case for the Draupner wave, along with the likewise famous Andrea rogue wave^39^, which has, however, a steeper front crest, which is slightly detached from the others profiles (Fig. 4). No specific relationship between the minimum elevation of troughs preceding and following *z*_m_ is observed. In the context of 3D wave fields, we note that, as the Draupner and Andrea waves were isolated as global maxima in a time record, it might also be that nearby the measurement point the sea surface elevations were even larger (as shown in the right panel of Fig. 3, and in Fig. 4 of Donelan and Magnusson^40^).

**Figure 5:**
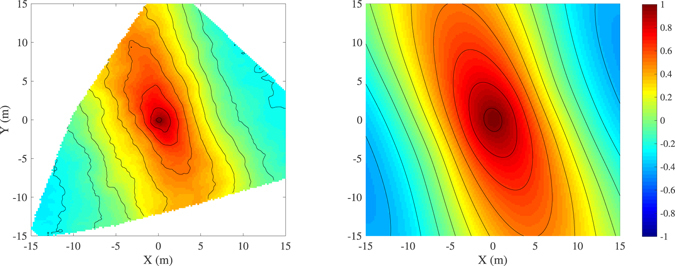
(left panel) Normalized average 3D shape of the measured sea surface elevation field at the time of the rogue waves collected within the record AA1. Only the rogue waves (10 out of 23) occurring close to the center of the observed region were used. (right panel) Normalized ST autocovariance function at the focusing time (*τ* = 0) computed using the directional wave spectrum of the sea state in the record AA1. The 3D fields are shown in the geographic reference system.

The existence of a predictable (in a stochastic sense) ST shape of high ocean waves is not novel *per se*. Indeed, the expected shape of large 3D wave groups can be estimated, for example, using the Slepian Model Representation^41^ or the Quasi-Determinism (QD) theory^42–44^, which, for a Gaussian wave field *z*_1_(*x*, *y*, *t*) with variance *σ* ^2^, state that the average shape *η*_1_ of the highest waves scales with the ST autocovariance function *ψ*(*X*, *Y*, *τ*) of *z*_1_(*x*, *y*, *t*). That is,

1 $${\eta }_{1}={z}_{{\rm{lm}}}\frac{{\psi }(X,\,Y,\,\tau )}{{\sigma }^{2}}$$

where *z*_1m_ is a large maximum of the sea surface elevation field, ***X*** = (*X*, *Y*) is the 2D horizontal vector and *τ* the time lag measured from the absolute maximum of *ψ*. An example of the average 3D shape of the rogue waves is shown in Fig. 5, where the observed shape is compared with the prediction based on the ST autocovariance function at the focusing time, i.e. *ψ*(*X*, *Y*, 0). Albeit the former is estimated with few realizations and the latter is valid for Gaussian fields, the empirical and the theoretical 3D shapes share common features (e.g., the short-crestedness), as observed by Forristall^45^ using numerical simulations.

At the spatial position (*X*, *Y*) = (0, 0) of the apex of the 3D group development, the autocovariance function is paired (via Fourier transform) to the omni-directional frequency spectrum by the following equation

2 $$\psi (\tau )={\int }_{0}^{\infty }S(\omega )\cos (\omega \tau ){\rm{d}}\omega $$

where *ω* = 2*πf* is the cyclic frequency, and we set *ψ*(*τ*) = *ψ*(0, 0, *τ*) for simplicity.

**Figure 6:**
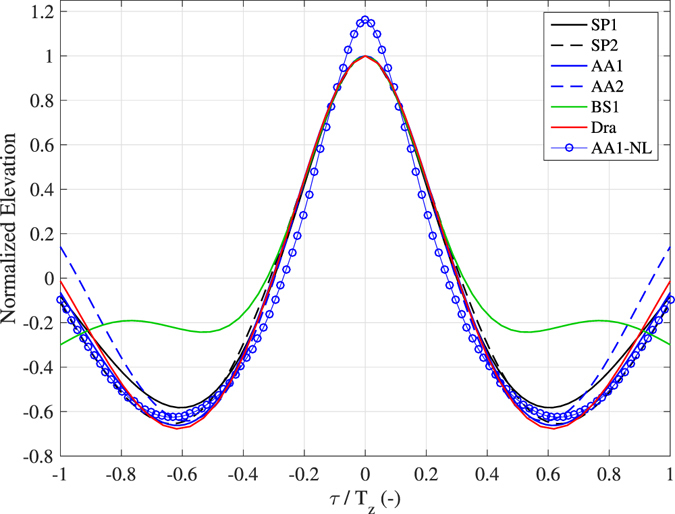
Average temporal profile of the highest waves. Results from the linear QD model for different sea conditions (Fig. 1 and Table 1). The second-order nonlinear profile for the record AA1 is also shown (AA1-NL).

For the sea states analyzed in this study, Fig. 6 shows the normalized linear profiles $\psi (\tau )/\psi (0)$, which are symmetric around the maximum elevation. Likewise to the observed profiles, for large elevations the theoretical profiles are very similar to each other. This result is expected for sea states with energies that present an equilibrium range, which would guarantee the Froude similarity between the sea states^43^. The presence of a secondary spectral mode seems not to influence the largest elevations, while it has an effect near the troughs (see the flatter troughs of the BS1 profile shown in Figs 4 and 6, and, for comparison, Figure 4.9 of the textbook by Boccotti^41^), in dependence on the position of the secondary peak with respect to the principal one. The theoretical shape given in Eq. (1) is distorted by the inclusion of higher-order harmonics, with a dominant contribution expected by the phase-locked second-order bound nonlinearities^7, 8^, which produce waves with higher and sharper crests and shallower more rounded troughs (Fig. 6). In the context of the QD model, the apparent asymmetry of the observed profiles shown in Fig. 4 might indicate that all the groups were not at the focusing point, although the limited extension of the sea surface covered by the stereo systems does not permit to draw a firm conclusion on this aspect. Analyzing the same stereo records collected near *Station P*, Schwendeman and Thomson^46^ observed an asymmetry near the crest of the breaking waves, that the authors connected to the crest tilting effect of waves prior to reaching the peak of the group.

### How likely are rogue waves in space and time?

Because the rogue waves described in the previous sections have been isolated from ST records, the question arises as to the occurrence probability of these extreme events and the appropriate statistical approach for describing their likelihood. The first implication of treating rogue waves as local maxima in space as well as in time is that, when examining a sea surface 2D region *Ω* wider than a single position *P* ∈ *Ω*, a larger number of rogue waves is found. This evidence can be inferred from the right panel of Fig. 3, in which the random positions where the sea surface elevation exceeds the rogue wave threshold 1.25*H*_s_ are highlighted: looking at the wrong place, we would not see any rogue wave. Indeed, watching for maxima over a 2D region we may relax the hypothesis of uniformity of the wave field. As a matter of fact, the actual short-crestedness (see for instance the large crest visible in the left panel of Fig. 3, and the 3D shape in Fig. 5) and the wavenumber/direction dispersion of the 3D wave groups make wave maxima larger and more numerous when they are sought over an area *A* > 0, rather than on a point^19, 47^. In other words, it is unlikely that unsteady 3D wave groups focus at (or close to) a specific point *P* ^19, 34^.

As a matter of fact, analyzing a long series of single-point field measurements, Christou and Ewans^2^ established that only one every 145 20-min sea states contained one rogue wave, implying that these waves occur rarely, on average once every about ∼33000 waves (a recent study by Gemmrich and Thomson^15^ revealed similar statistics analyzing time records at *Station P*), consistent with the Tayfun model^8^ prediction of the probability that nonlinear crest heights exceed 1.25*H*_s_. In this respect, a key finding of our study is that at least one wave whose crest height exceeds 1.25*H*_s_ was observed within the ST records, which contain on average less than 12000 individual 3D waves (Table 1), the minimum (*N*_3D_ ~ 400–600) being for the two records collected near *Station P*. The actual number of rogue waves is even higher, as we expect that more than one rogue event occurred within each ST record. Indeed, in a thorough analysis of the record AA1, Benetazzo *et al*.^47^ revealed as many as 23 rogue events belonging to independent 3D waves over a ST region containing on average 10830 waves: one rogue event every ~ 500 waves.

**Figure 7:**
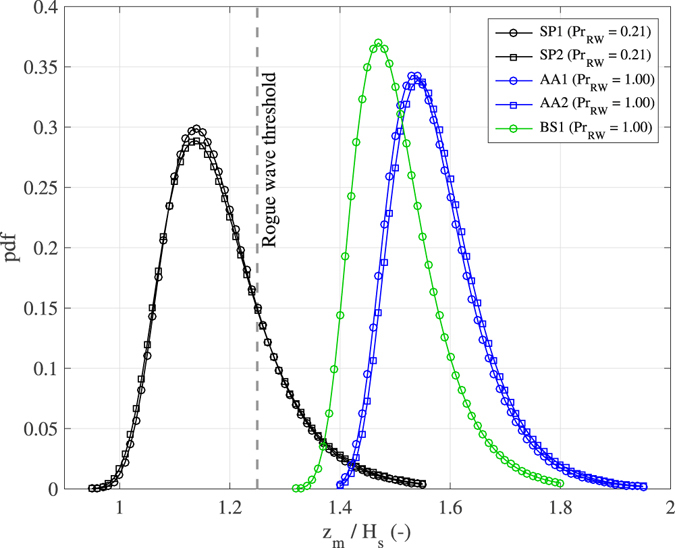
Theoretical probability density function (pdf) of ST extreme second-order nonlinear crest heights. In the legend, *Pr*_RW_ = Pr{max{*z*(*x*, *y*, *t*)} > 1.25*H*_s_ | (*x*, *y*) ∈ *Ω*, *t* ∈ *D*} is the probability that the maximum crest height over the ST region *AD* exceeds the rogue threshold *z*_m_ = 1.25*H*_s_ (shown by the dashed gray line). We note that the presence of a breaking-limited maximum crest height (e.g., 1.7*H*_s_ as suggested by Donelan and Magnusson)^40^ is not included in the theoretical model.

In search for a short-term statistics explaining the rogue waves observed within the ST records, we rely on the study of Fedele^35^, who derived an asymptotic extreme value distribution (based on the Euler Characteristic approach)^48^ of maximum crest heights occurring in a ST Gaussian field. This topic has been investigated also by Piterbarg^36^, whose method was successfully applied to the estimation of maximum wave crests^45, 49^. For weakly nonlinear random seas dominated by second-order nonlinearities, the Fedele’s solution was extended by Benetazzo *et al*.^47^ using the Tayfun^8^ model. This theoretical approach was validated using the sample of 23 rogue waves taken from the record AA1. For the five sea states considered here, we show in Fig. 7 the Gumbel-like probability density function (pdf) fitting the ST extreme nonlinear model by Benetazzo *et al*.^47^. We note that each observed crest height *z*_m_ lies within the 99% confidence interval of the respective distribution.

The implication of the high (and larger than 1.25*H*_s_) modal values of the pdfs for records AA1, AA2, and BS1 is that, in those sea conditions, the probability to find a rogue wave within one realization of the ST record would be practically equal to 1. For the records SP1 and SP2 the probability would be smaller than 1, about 0.2, as a result of a relatively small area *A* compared to the average wave and crest lengths. These theoretical probabilities of rogue wave occurrence are considerably high, at least one order of magnitude larger than the ones obtained excluding the spatial contribution (i.e. imposing *A* = 0) in the distribution of the random variable “maximum surface elevation”.

The physical mechanism underling the distribution of ST extremes and thus contributing to the formation of rogue waves is the constructive interference of elementary waves^50^, with a significant role played by the wave dispersion^51^. The coming into phase of different frequency components responsible of localized dispersive focusing has already been recognized^2, 52^ as a mechanism responsible for rogue wave generation in generic storm conditions. The fundamental enhancement due to nonlinearities^53^ is limited to the contribution of second-order bound harmonics, as the effect of third-order nonlinearities is expected to be small^1, 54^, and thus the role of the modulation instability is limited.

### Sea state conditions

This section discusses whether the temporal profile and the distribution function of rogue waves are linked to the sea condition at the time of their acquisition. The study of a deterministic model connecting the rogue wave development to the sea state conditions has not been fully explored. In the context of rogue wave generation through nonlinear focusing due to third-order quasi-resonant wave-wave interactions, Waseda *et al*.^55^ found that during freakish sea states the directional spreading is narrower. However, Fedele *et al*.^1^ justified oceanic rogue waves excluding the nonlinear focusing, and Cavaleri *et al*.^3^ suggested the presence of two large crossing wave systems being responsible for a large probability of occurrence for rogue waves at the time of the Draupner event. On the top of this, Christou and Ewans^2^, analyzing a large dataset of measurements, concluded that the existence of rogue waves within a time record is not governed by the sea state (such as the nonlinear parameters steepness, skewness, and kurtosis) or environmental conditions (such as the directional spreading).

To inspect this aspect of the problem, we examine the characteristic sea state parameters of the ST wave fields (Table 2). The observations include seas with mean wave steepness *μ* (computed as in Fedele and Tayfun^56^), ranging from low (*μ* = 0.04 for the record BS1) to mild values (*μ* = 0.06 for the records AA1 and AA2). The bandwidth parameter *ν* ^57^ embraces values typical of active sea conditions, around 0.50 for all records. The sea states have energies differently distributed over directions: the one-sided directional spreading *σ*_*θ*_ is smaller than 30° for records SP1 and AA1, whilst the largest spreading *σ*_*θ*_ = 66° is for the record BS1. All the sea states were indeed short-crested, having ~1 the short-crestedness parameter *γ*_s_^58^, which we interpret as the ratio between spreading of the waves in the wavenumber domains *k*_y_ and *k*_x_ (assuming the *x*-axis coincident with the mean direction of wave propagation, and the *y*-axis orthogonal to it).

**Table 2:** Sea state parameters during the stereo acquisitions, and for the Draupner event.

	*μ*	*ν*	*σ*_*θ*_	*γ*_s_	*λ*_3_	*λ*_4_
SP1	0.05	0.48	27°	0.84	0.08	3.05
SP2	0.05	0.50	42°	0.91	0.09	3.27
AA1	0.06	0.50	27°	0.93	0.16	3.22
AA2	0.06	0.44	46°	0.93	0.16	3.14
BS1	0.04	0.57	66°	1.05	0.08	3.03
Draupner	0.07	0.49	25°	0.72	0.41	4.07

Mean spectral wave steepness: *μ*; spectral bandwidth parameter: *ν*; one-sided directional width: *σ*_*θ*_; short-crestedness parameter: *γ*_s_; skewness coefficient: *λ*_3_; kurtosis coefficient: *λ*_4_.

The effect of second-order and third-order nonlinearities on the likelihood and shape of rogue waves can be quantified using the skewness (*λ*_3_) and kurtosis (*λ*_4_) coefficients. In particular, *λ*_3_ describes the effects of second-order bound nonlinearities, and *λ*_4_ include the dynamic component due to third-order nonlinear interactions and a bound contribution^8, 11^. Among the ST records, a large variability is found for both these parameters: the maximum value of the skewness *λ*_3_ = 0.16 is for records AA1 and AA2 (for which we should expect a larger effect of second-order nonlinearities), while the maximum contribution of third-order nonlinearities would be expected during the record SP2 (for which *λ*_4_ = 3.27). We note that large skewness and kurtosis coefficients for the Draupner event (reported in Table 2) are misleading, as the evaluation of both coefficients is biased by the presence of a very high wave in a single time record^59^.

A major contribution to the pdf of ST extreme nonlinear crest heights is given by the steepness of the sea state: the larger *μ*, the less peaked the pdf (Fig. 7). As a matter of fact, for a given pdf of linear ST extremes, the density function of nonlinear ST extremes is more narrowed around its modal value in those sea states for which the effect of nonlinearities is expected to be smaller. On the other hand, strong nonlinear conditions spread extremal waves over a wider range, and, as it is expected, shift crest heights towards larger values, making rogue waves more likely. In this, our results integrate the study by Christou and Ewans^2^, as these authors limited the analysis to time records of sea surface elevations.

However, examining the rogue wave temporal shapes (Fig. 4), we note that they seem to be marginally influenced by the characteristics of the sea state, as if, once interfering constructively in frequency and direction, the elementary waves (enhanced by nonlinearities) produce a well defined profile of rogue waves (which should hold also for any large wave, as the threshold 1.25*H*_s_ is of course arbitrary).

## Conclusions

In this study, we have examined a set of five space-time records of sea surface elevations in search of rogue waves. The motivation is twofold: (1) to analyze the shape of the rogue waves, once they are isolated as maxima of 3D unsteady groups; (2) to discuss how likely these extreme waves are, when looking in space as well in time. This research presents an experimental evidence that the temporal profile of rogue waves tends to follow a general shape, which, once scaled with the severity of the sea state, results to be slightly dependent on the sea state conditions. The constructive interference of dispersive elementary 3D waves enhanced by the interaction with the bound modes seems to be effective in explaining this behavior.

Not surprisingly, within a space-time wave field, rogue waves are on average more numerous than in single-point time series (of the same duration). However, in space-time records rogue waves are even more likely than in time records, as within each space-time field considered in this study at least one rogue wave was detected. We argue that the large probability of occurrence is the result of the wavenumber/direction dispersion, which concentrates the highest waves in small regions of the sea surface. As a result, the probability of gathering rogue waves in space and time is at least one order of magnitude larger than the probability restricting the analysis to time only. This fact may be determined also from the exceedance distribution given in Eq. (9), comparing the temporal 1D term, proportional to *N*_1_, and the spatio-temporal 3D term, proportional to *N*_3_(*ζ* ^2^ − 1).

In the study of rogue waves, longer series of space-time wave data will be helpful for a thorough characterization of the long-term occurrence of extreme waves, which has implications, for example, for the evaluation on ships and offshore structures (which occupy always a footprint *A* > 0) of the impact with rogue waves, which are presently neglected by the classification rules and offshore standards (e.g., Bitner-Gregersen and Gramstad)^60^.

## Materials and Methods

### Stereo image processing

The five ST records of sea surface elevations were collected using three different stereo wave imaging systems. The first system was installed on the *Acqua Alta* oceanographic research platform (the local depth is 17 m) in the northern Adriatic Sea (Italy; 45.32°N, 12.51°E), and it relies on two BM-500GE JAI digital cameras mounting 5-mm focal length lenses^23^. Images were acquired at a frame-rate of 15 Hz (record AA1) and of 12 Hz (record AA2). Details of the stereo pipeline and the post-processing strategy of wave data can be found in the studies of Benetazzo *et al*.^47, 61^. An example of the ST evolution of the wave field can be seen in the Supplementary Video S1.

A second stereo video system was deployed aboard the R/V *Thomas G*. *Thompson* during a cruise to *Station P* in the North Pacific (50.1°N, 144.9°W; depth ~ 4200 m). Cameras consisted on two PointGrey Flea2 with 9-mm focal length lenses, used in Schwendeman and Thomson^46^ for estimating whitecap coverage. Stereo cameras’ position and rotation with respect to the geographic reference was determined using a Novatel combined inertial motion unit and a global navigation satellite system. Images were acquired at a frame-rate of 5.0 Hz (record SP1) and of 7.5 Hz (record SP2). A thorough description and validation of the stereo wave measurements collected from the vessel is performed in Schwendeman and Thomson^62^.

The third system was mounted on the research platform of the *Marine Hydrophysical Institute* off the coast next to Katsiveli in the Black Sea (44.39°N, 33.98°E), near the southern tip of Crimea. The water depth at the observation area is about 30 m. It relies on same cameras and lenses as in the *Acqua Alta* experiment. Stereo images were analyzed with the algorithm described in Leckler *et al*.^63^, an improved version of that one used by Benetazzo *et al*.^23^.

After the stereo processing and before the analysis of the maximum elevations, scatter 3-D data were linearly interpolated on a spatial grid (326 × 426 points in AA1 and AA2; 161 × 161 points in SP1 and SP2; 193 × 198 points in BS1) with uniform *xy*-resolution (0.20 m in AA1 and AA1; 0.25 m in SP1 and SP2; 0.10 m in BS1). Then, the time series at each grid point of the region *Ω* were filtered by a 2-Hz (records AA1, AA2, and BS1) or 1-Hz (records SP1 and SP2) Butterworth filter to reduce the influence of the high-frequency noise.

### Sea state parameters and distribution of ST wave extremes

The severity of the sea state recorded by the stereo system is defined using the significant wave height, denoted by *H*_s_ and evaluated as *H*_s_ = 4*σ*, where *σ* is the standard deviation of *z*(*x*, *y*, *t*). The average zero-crossing period *T*_z_ is equal to $\sqrt{{m}_{0}/{m}_{2}}$, where ${m}_{{\rm{i}}}={\int }_{f}{f}^{{\rm{i}}}S(f){\rm{d}}f$ is the *i*-th order moment of the frequency spectrum *S*(*f*), which is computed via Fourier transform of the time series *z*(*t*) holding the maximum sea surface elevation *z*_m_. The bandwidth *ν* ^57^ of *S*(*f*) is defined as $\nu =\sqrt{{m}_{0}{m}_{2}/{m}_{1}^{2}-1}$.

The directional spectrum *S*(*f*, *θ*) of the records AA1, AA2, and BS1 was computed using the Extended Maximum Entropy Principle method (EMEP)^64^ applied to time series randomly chosen within the region *Ω*. The frequency – direction spectrum was resolved with 180 equally spaced directions to cover the full circle and 1024 uniformly distributed frequencies from 0.05 to 2.00 Hz. During the acquisitions SP1 and SP2, *in situ* measurements of the local wave spectrum were made using Datawell DWR-G4 directional Waverider buoys. The spectral calculations were performed over 30-minute intervals using Datawell’s built-in processing^65^, over 64 frequencies (0.025 Hz to 0.580 Hz) and 90 equally spaced directions.

The one-sided directional width *σ*_*θ*_ of the spectrum *S*(*f*, *θ*) is computed with respect to the peak direction as in the textbook by Holthuijsen^66^. To limit the influence of instrumental noise, in the computation of *σ*_*θ*_ we set equal to zero the wave energies smaller than 1/100 the maximum energy. In analogy with the definition of the directional spreading, in the spatial domain (*x*, *y*) the short-crestedness (→ 0 for very long-crested waves) *γ*_s_ = *L*_x_/*L*_y_ expresses the ratio between the standard deviations of *S*(*f*, *θ*) along the wavenumber *y*-axis and *x*-axis, respectively, and it is computed as

3 $${\gamma }_{s}=\sqrt{\frac{{m}_{020}}{{m}_{200}}}$$

where ${m}_{{\rm{i}}{\rm{j}}{\rm{l}}}=\iint {k}_{x}^{i}{k}_{y}^{j}{f}^{l}S(f,\theta ){\rm{d}}f{\rm{d}}\theta$ is the *ijl*-th order moment of the directional spectrum *S*(*f*, *θ*).

The coefficients of skewness (*λ*_3_) and kurtosis (*λ*_4_) have been estimated from the sea surface elevation field *z* = *z*(*x*, *y*, *t*) applying the following formulae:

4 $${\lambda }_{3}=\frac{ < {(z- < z > )}^{3} > }{{\sigma }^{3}}$$

5 $${\lambda }_{4}=\frac{ < {(z- < z > )}^{4} > }{{\sigma }^{4}}$$

where the angle brackets < > denote the ensemble average.

Using the directional wave spectrum, the ST autocovariance function is estimated as follows:

6 $$\psi ({\boldsymbol{X}},\tau )={\int }_{0}^{\infty }{\int }_{-\pi }^{\pi }S(\omega ,\theta )\cos ({\boldsymbol{k}}\cdot {\boldsymbol{X}}-\omega \tau ){\rm{d}}\omega {\rm{d}}\theta $$

where ***k*** = (*k*_x_, *k*_y_) = (*k*cos*θ*, *k*sin*θ*) is the wavenumber vector associated with the cyclic frequency *ω* and direction *θ* via the linear dispersion relation for gravity waves.

The ST extreme value distribution of nonlinear crest heights was fitted with a Gumbel distribution, whose scale parameters *β* = 1/*α* and mode *h* were determined as follows^47^:

7 $$h=({h}_{1}+\frac{\mu }{2}{h}_{1}^{2})$$

8 $${\rm{\alpha }}=({h}_{1}-\,\frac{2{N}_{3}{h}_{1}+{N}_{2}}{{N}_{3}{h}_{1}^{2}+{N}_{2}{h}_{1}+{N}_{1}})/(1+\mu {h}_{1})$$

where *μ* is a measure of the mean wave steepness^56^, and *h*_1_ is the mode of the probability density function of ST extreme linear crest heights *z*_1m_, whose probability of exceedance is approximated (for large values of the threshold $\zeta$) by the following equation^35^

9 $$Pr\{{z}_{1{\rm{m}}} > \zeta \sigma \}=[{N}_{3}(\zeta {}^{2}-1)+N{}_{2}\zeta +{N}_{1}]\exp (-{\zeta }^{2}/2)$$

The coefficients *N*_3_, *N*_2_, and *N*_1_ are proportional to the average number of waves within the 3D space-time volume, on its 2D lateral faces, and on its 1D edges, respectively^35, 67^.

## Electronic supplementary material

Example of 3D wave fields
